# STOP: an open label crossover trial to study ICS withdrawal in patients with a combination of obesity and low-inflammatory asthma and evaluate its effect on asthma control and quality of life

**DOI:** 10.1186/s12890-022-01843-0

**Published:** 2022-02-05

**Authors:** Jan A. Witte, Gert-Jan Braunstahl, Wouter J. B. Blox, Susan C. van ’t Westeinde, Johannes C. C. M. in ’t Veen, Jasper H. Kappen, Elisabeth F. C. van Rossum

**Affiliations:** 1 Department of Pulmonology, STZ Centre of Excellence for Asthma and COPD, Franciscus Group, Rotterdam, The Netherlands; 2 Department of Pulmonology, Erasmus MC, University Medical Center Rotterdam, Rotterdam, The Netherlands; 3 Department of Pulmonology, Albert Schweitzer Ziekenhuis, Dordrecht, The Netherlands; 4 Department of Pulmonology, Maasstad Ziekenhuis, Rotterdam, The Netherlands; 5 Allergy and Clinical Immunology, Immunomodulation and Tolerance Group, Inflammation Repair and Development, Imperial College, National Heart and Lung Institute, London, UK; 6 Department of Internal Medicine, Division of Endocrinology, and Obesity Center CGG (Centrum Gezond Gewicht), Erasmus MC, University Medical Center Rotterdam, Rotterdam, The Netherlands

**Keywords:** Asthma, Obesity, T2-low, Corticosteroids, ICS tapering, RCT

## Abstract

**Background:**

Asthma patients with obesity often have a high disease burden, despite the use of high-dose inhaled corticosteroids (ICS). In contrast to asthmatics with normal weight, the efficacy of ICS in patients with obesity and asthma is often relatively low. Meanwhile, patients do suffer from side effects, such as weight gain, development of diabetes, cataract, or high blood pressure. The relatively poor response to ICS might be explained by the low prevalence of type 2 inflammatory patterns (T2-low) in patients with asthma and obesity. T2-low inflammation is characterized by low eosinophilic count, low Fractional exhaled NO (FeNO), no clinically allergy-driven asthma, and no need for maintenance oral corticosteroids (OCS). We aim to study whether ICS can be safely withdrawn in patients with T2-low asthma and obesity while maintaining an equal level of asthma control. Secondary outcomes focus on the prevalence of ‘false-negative’ T2-low phenotypes (i.e. T2-hidden) and the effect of ICS withdrawal on parameters of the metabolic syndrome. This study will lead to a better understanding of this poorly understood subgroup and might find new treatable traits.

**Methods:**

The STOP trial is an investigator-initiated, multicenter, non-inferiority, open-label, crossover study aiming to assess whether ICS can be safely withdrawn in adults aged 17–75 years with T2-low asthma and obesity (body mass index (BMI) ≥ 30 kg/m^2^). Patients will be randomly divided into two arms (both n = 60). One arm will start with fixed-dose ICS (control group) and one arm will taper and subsequently stop ICS (intervention group). Patients in the intervention group will remain ICS naïve for ten weeks. After a washout of 4 weeks, patients will crossover to the other study arm. The crossover study takes 36 weeks to complete. Patients will be asked to participate in the extension study, to investigate the long-term metabolic benefits of ICS withdrawal.

**Discussion:**

This study yields valuable data on ICS tapering in patients with T2-low asthma and obesity. It informs future guidelines and committees on corticosteroid-sparing algorithms in these patients.

*Trial registration* Netherlands Trial Register, NL8759, registered 2020–07-06, https://www.trialregister.nl/trial/8759.

**Protocol version and date:** version 2.1, 20 November 2020.

## Background

Asthma is a highly prevalent chronic pulmonary disease characterized by wheezing, shortness of breath, and cough. Asthma has a significant disease burden [1] and is considered an umbrella diagnosis for several phenotypes and endotypes. Endotypes may broadly be described as T2 high and T2 low asthma, dependent on the inflammatory mechanisms that drive the disease. Patients with asthma and obesity represent a phenotype frequently characterized by the absence of type 2 inflammation. In these patients, evidence for increased activation of central type 2 inflammatory cells is usually absent. The lack of type 2 inflammation (i.e. T2 low asthma) may explain the poor response to corticosteroids in patients with asthma and obesity [2–6], as central type 2 inflammatory cell types, such as T-helper 2 cells and eosinophils are major targets for corticosteroids and biologicals [7]. Nevertheless, almost all asthma patients are treated with inhaled corticosteroids (ICS), as recent guidelines advocate ICS as a cornerstone of asthma treatment without any distinction of the various asthma phenotypes [8]. As a result, patients with T2-low asthma and obesity are exposed to the side effects of corticosteroids without experiencing positive effects on their asthma symptoms. Side effects include adrenal insufficiency, cardiovascular disease, enhanced appetite, weight gain, hyperglycemia, osteoporosis, neurocognitive symptoms, and an increased risk of infectious disease [9–14].

These side effects can be prevented by minimizing ICS dosage. Dose reduction has been studied before, initially to induce asthma exacerbations and later to study dose optimization algorithms, but did not result in a successful ICS discontinuation strategy [15–22]. However, important clues were reported. For example, ICS tapering was better tolerated in patients with low blood or sputum eosinophils, suggesting lower ICS efficacy in patients with T2-low asthma [18–21]. A recent study by Heaney et al. used a dose guiding algorithm including blood eosinophils, Fractional exhaled NO (FeNO) and/or Periostin to adapt ICS dose [18–22]. This algorithm successfully reduced the cumulative dose of ICS when compared to usual care. However, complete ICS withdrawal was achieved in just 5% of all patients: these patients had a T2-low asthma [22]. Therefore, low success-rates of complete ICS withdrawal might be contributed to the high prevalence of T2-high asthma in prior studies. Therefore, to study ICS withdrawal, a clear distinction on T2 endotype should preselect patients with low ICS efficacy.

### Objectives

In this trial, we withdraw ICS in patients with T2-low asthma and obesity to determine the effects on asthma control. We hypothesize that ICS withdrawal is non-inferior to ICS continuation in patients with asthma and obesity. Additionally, this trial uses common T2-biomarkers to detect patients that become T2-high after ICS withdrawal; T2-hidden asthma.

## Methods

### Study design

The STOP trial is initiated by the ‘STZ center of Excellence for Asthma and COPD’ of the Franciscus Gasthuis & Vlietland hospital, collaborating with the Maasstad hospital and the Albert Schweitzer hospital. This multicenter approach ensures a larger number of possible study subjects and decreases selection bias due to social-economical differences between regions of Rotterdam. Patients will be randomized into one of two study groups to avoid allocation bias. Both groups consist of a control period and an intervention period in a 2 × 2 crossover pattern that creates balanced groups maximizes statistical power, and allows for analysing a possible sequence effect. The study has a non-inferiority design, which tests the hypothesis that ICS tapering is not inferior to ICS continuation. This intervention trial has an open-label to determine the effects of ICS tapering in a real-life situation.

### Population

**Fig. 1:**
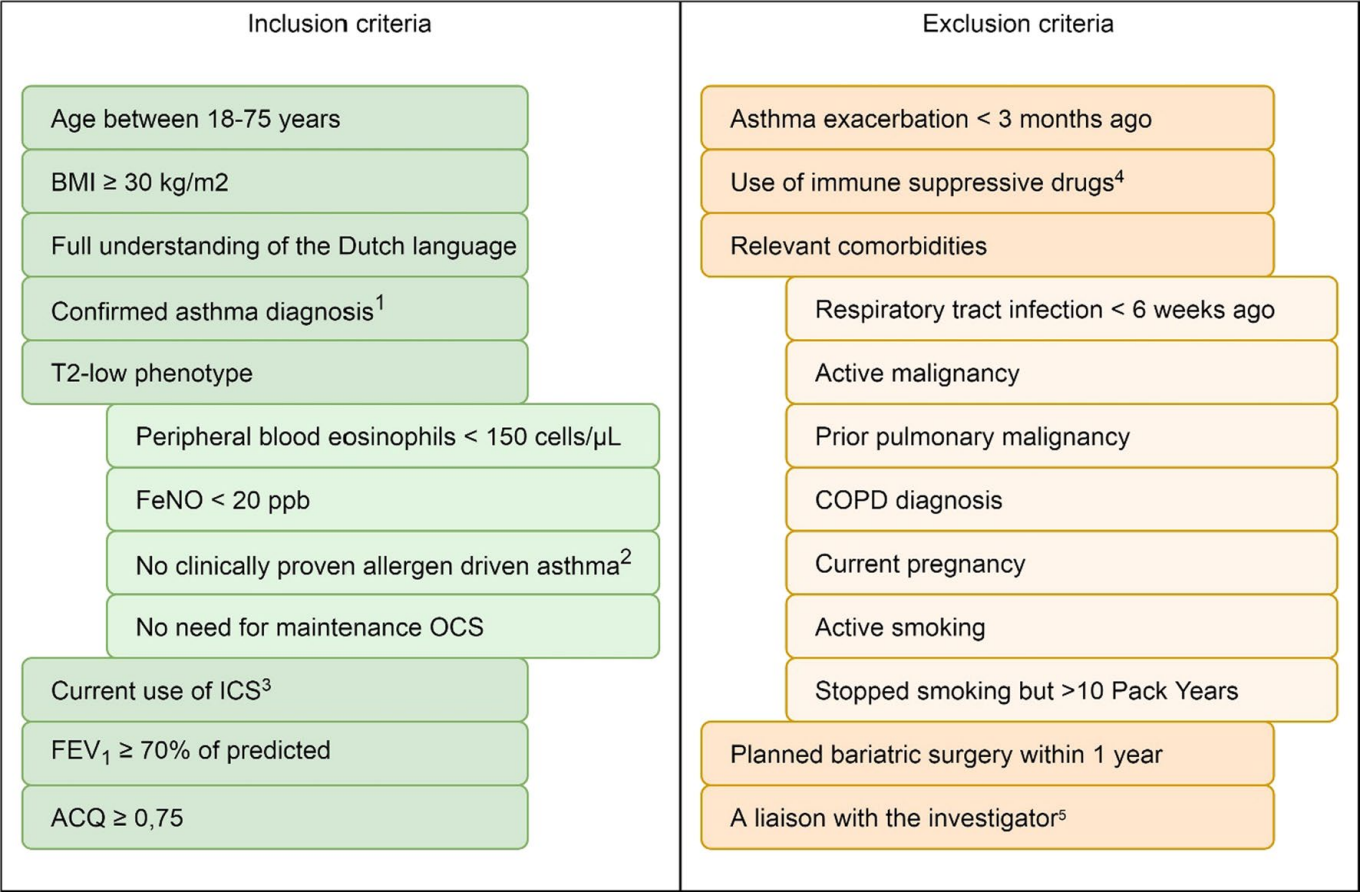
Overview of in- and exclusion criteria. 1) 12% reversibility in FEV1 or positive histamine/methacholine provocation test. 2) Allergic sensitization as proven by a skin prick test or specific IgE in combination with clinically relevant symptoms triggered by culprit allergen (Ansotegui, WAO position paper 2020). 3) 400–800 mcg beclomethasone daily (or dosisequivalent of other ICS). 4) Immune suppressive drugs, such as biologicals/monoclonal antibodies, calcineurine inhibitors, mTOR inhibitors and IMDH inhibitors. 5) in concordance with the Medical Research Involving Human Subjects Act (Dutch: WMO—article 5). (*BMI* Body Mass Index, *FeNO* Fractional exhaled Nitric Oxide, *OCS* Oral CorticoSteroids, *ICS* Inhaled CorticoSteroids, *FEV*_1_ Forced Exhaled Volume in 1 s, *ACQ* Asthma Control Questionaire, *COPD* Chronic Obstructive Pulmonary Disease)

Adults aged between 18 and 75 years with a Body Mass Index (BMI) ≥ 30 kg/m^2^ and a confirmed T2-low asthma diagnosis will be recruited at the outpatient clinic of the pulmonary departments of three major hospitals in the region of Zuid-Holland, the Netherlands; Franciscus Gasthuis & Vlietland hospital (Rotterdam), Maasstad hospital (Rotterdam), and Albert Schweitzer hospital (Dordrecht). In- and exclusion criteria must be met before the start of the run-in period. A detailed description of the in- and exclusion criteria can be found in Fig. 1.

### Randomization and blinding

Patients included in the study will be randomized into two groups: the first group with sequence Intervention-Control and the second group with sequence Control-Intervention. Randomization is performed by Castor Data Capturing software using variable block randomization with block sizes of 4/6/8 and stratification by study centre.

### Study procedures

**Fig. 2:**
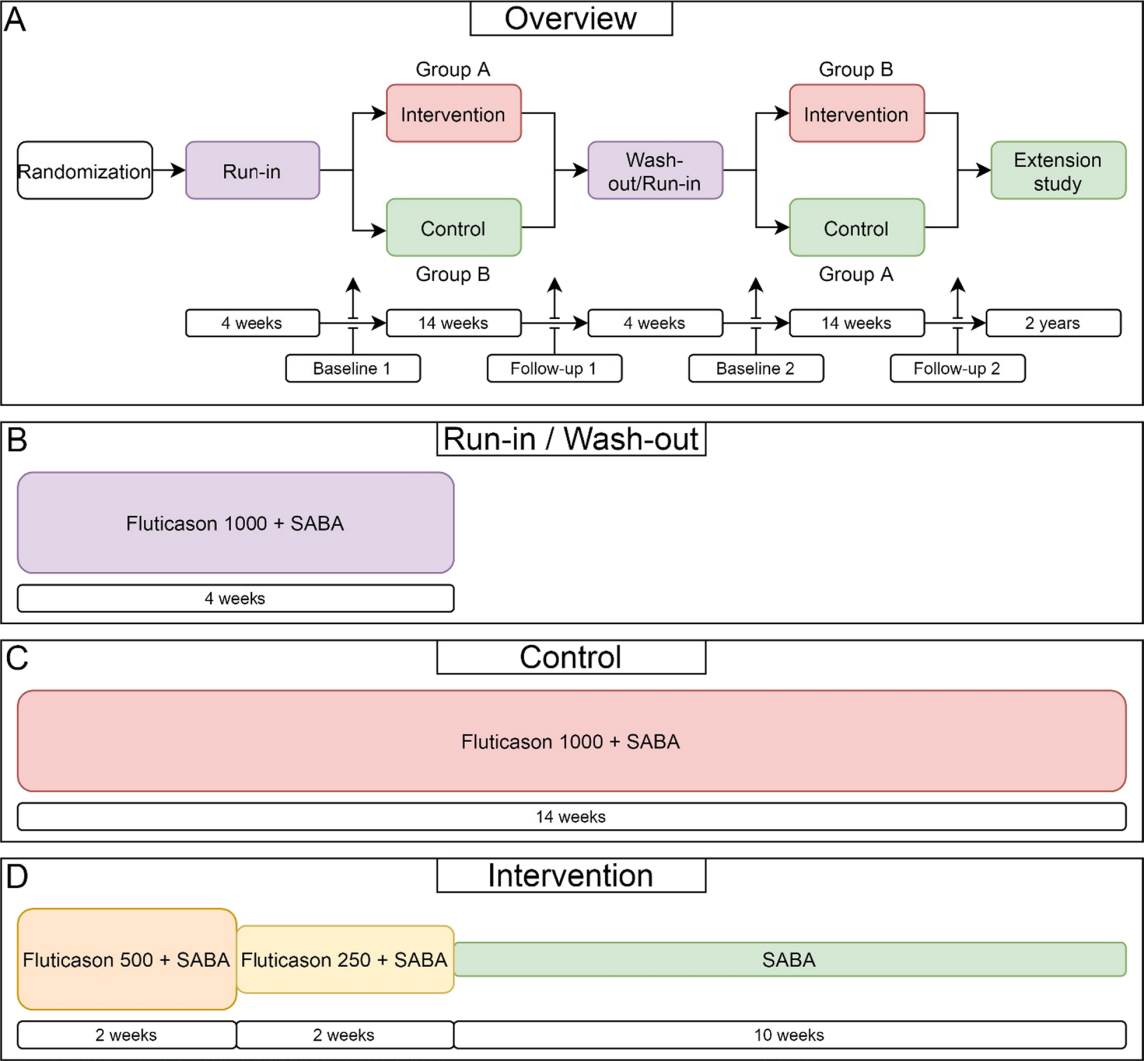
**A** Schematic overview of the STOP trial: a 4 week run-in, followed by either an intervention period or a control period of 14 weeks, followed by a crossover. After completion of both treatment periods, patients may choose to participate in the extension study. **B** All patients will be enrolled with a prescription of Fluticasone 1000 mcg and Salbutamol 100 ug (as needed). **C** Patients receive a stable dose of ICS during the control period. **D** During the intervention period, patients half the dose of Fluticasone every 2 weeks. Patients will discontinue ICS after 4 weeks and remain ICS naïve for 10 weeks

All patients start with a run-in period of 4 weeks using a fixed dose of ICS (Fluticasone 1000mcg/day) as well as salbutamol 100mcg ‘as needed’ (see Fig. 2 and Table 1). This will equalize baseline ICS-exposition. (The period of 4 weeks is similar to earlier tapering studies [15–21]).

**Table 1:** Overview of procedures during STOP trial. These procedures will be used in both crossover period 1 and crossover period 2

	Time (weeks)	− 2	0 and 18	4 and 22	6,8,13 and 24,26,31	18 and 36
	Period (1 or 2)	1	1 and 2	1 and 2	1 and 2	1 and 2
	Visit type	Inclusion	Run-in	Baseline	Follow up	Follow up
Activity/Assesment						
Live		x	x	x		x
Telephone					x	
Check inclusion criteria		x				
Informed consent		x				
Medication check		x				
Prior medical history		x				
Comorbidities		x				
Anthropometrics		x		x		x
Exacerbation frequency		x	x	x	x	x
Questionnaires		x	x	x	x	x
Blood samples				x		x
Scalp Hair				x		x
Lung function test*			x	x		x
eNose				x		x
Study information		x	x	x	x	x
Prescriptions			x	x	x	x
SAE form		Thoughout study				

After the run-in period, patients in arm 1 will discontinue ICS (intervention), while patients in arm 2 will continue with a fixed-dose ICS for 14 weeks (control). An abrupt stop of ICS might cause asthma flair-ups. Therefore, ICS-dose will be tapered in two steps (fluticasone 500mcg/daily and 250 mcg/daily) over 4 weeks (see Fig. 1). After those 4 weeks, patients will become ICS-naïve for 10 weeks. Similar studies (which typically had a mix of both T2-high and T2-low patients) used an ICS naïve duration between 20 days and 10 weeks, of which 8 weeks and 10 weeks were most commonly used [15–21]. Empty canisters of salbutamol or fluticasone will be collected and weighed to evaluate protocol adherence.

After completing the first period, patients will crossover from the intervention arm to the control arm and vice versa. There is a-4 week washout/run-in period between the first and second periods to avoid carry-over effects. A second baseline ensures analysis of sequence effects. After completion of the second intervention period, intervention- and control data will be compared. The trial results will be shared with the participants and other relevant parties during a ‘patient participation meeting’.

This design is powered on demonstrating sustained asthma control, with a limitation in terms of measuring long-term health benefits (e.g. metabolic alterations) of ICS tapering. To study the long-term effects of ICS withdrawal in T2-low asthma patients with obesity, we will extend the study by 2 years in an open-label extension study. In this extension study, patients will remain ICS naïve. Patients may only participate in the extension study after successful discontinuation of ICS in the crossover period.

### Outcomes

#### Primary endpoint

The primary parameter is the ACQ-5, which is widely used in the Netherlands to monitor asthma control. The difference between the control and intervention periods will be analysed using the baseline ACQ-5 score as a reference [23].

#### Secondary study parameters

**Anamnestic parameters**

- Exacerbation frequency (mild/intermediate/severe)
- Medication
- Smoking status and exposure to drugs/vapors/fumes/gases/other chemicals
- Social-economic status
- Age at asthma diagnosis
- Co-morbidity (such as hypertension, OSAS, cardiovascular disease, stroke, diabetes, kidney failure, gastrointestinal reflux, malignancy, osteoporosis)
- Birth weight and respiratory history during childhood
- Adverse events
- Adherence to treatment regimen (using the weight of the inhaler at study visits)

**Questionnaires**

- **ACQ-5:** In this study the ACQ-5 will be used, consisting of five patient self-reported questions. The recall time is one week with a score range of 0 to 6 (0 = never and 6 = always). A higher score is associated with worse asthma control. The minimal clinically important difference of the ACQ is 0.5 [23].
- **AQLQ:** The AQLQ consists of 32 items with a 3 week recall period. The score range per question is 1 to 7 (1 = severely impaired and 7 = not impaired at all). It is used to qualify the disease-specific health-related quality of life [24–26].
- **MRC:** The MRC (Medical Research Council) is a dyspnea scale, as a measure of disability in patients with respiratory disabilities. It consists of one question about the shortness of breath and six possible answers. The score range per question is 0 to 5 (0 = no shortness of breath at all and 5 = too breathless to leave the house). A higher score is therefore associated with a high burden of disease [27].
- **FSS:** The FSS (Fatigue Severity Scale) measures fatigue severity. It has 9 items with are scored on a 7 point Likert scale. A higher score is associated with higher fatigue severity [28].

**Anthropometrics**

- Body Mass Index (BMI)
- Waist circumference
- Blood pressure

**Blood tests**

- **Metabolic and endocrine panel:** Oral corticosteroids are none for their dysregulating effects on glucose homeostasis [29]. This dysregulation was not seen in retrospective studies [30]. However, prospective studies comparing asthma patients with and without ICS have not been performed recently, as guidelines recommend ICS in all patients [31–33]. The design of this study fits the requirements for such an analysis. The following parameters will be tested at baseline, intervention period and control period: fasting glucose/insulin, morning cortisol, lipid spectrum, and liver function tests.
- **Pulmonary panel:** Inhaled corticosteroids suppress T2-high inflammatory pathways. Previous ICS tapering studies (mainly including patients with T2-high asthma) demonstrated increased blood eosinophils after ICS tapering or even used an ICS dosing algorithm based on blood eosinophils [15, 22]. However, these studies had no differentiation between T2-high and T2-low asthma. This trial solely includes patients with T2-low asthma during treatment with ICS and suspect these pulmonary parameters to remain low after ICS withdrawal. However, we postulate the presence of T2-hidden phenotypes, in which pulmonary biomarkers increase after ICS tapering. To study this phenomenon, a pulmonary panel will be tested, including blood eosinophils, total IgE and hematologic parameters.
- **Blood Serum:** Blood serum will be isolated and frozen at − 80 °C for analyses of inflammatory and endocrine biomarkers.
- **Isolation of Peripheral Blood Mononuclear Cells (PBMCs)**: To study the effect of ICS withdrawal on T2-low inflammatory cells.

**Lung function**

- **Expiratory flow assessment:** A hand-held spirometer will be used to assure maintained lung function after ICS withdrawal by measuring the FEV1.
- **FeNO:** Patients in this study have a starting FeNO lower than 20 ppb. However, we postulate that the FeNO values may increase to a value larger than 20 after ICS withdrawal. These patients have become T2-high (e.g. T2-hidden), which is an important secondary outcome.
- **eNose:** The electronic Nose is a novel development that uses semiconductors to measure patterns of exhaled volatile compounds. A eNose test results in a so-called ‘breath print’. The eNose successfully predicted response to (lung-)cancer treatment and is also used to measures the effect of corticosteroids in patients with Asthma [34–37]. We aim to study the relationship between breath print and treatment response to predict successful ICS withdrawal. Furthermore, the eNose might be able to predict the response to ICS in ICS naïve patients with newly diagnosed asthma.

**Other**

- **Hair cortisol and cortisone:** As parameters of long-term endogenous corticosteroid exposure.
- **Empty canisters:** To study treatment adherence.

### Data management

Data will be stored in an electronic database (Castor EDC) using the participant study number. A master file is available to link the study number with the corresponding patient number at every site. This master file is only accessible for the site investigators. Paper questionnaires and other relevant materials will be safely stored in a locked room. The material will be labelled with a study number, study site, date, and visit number. Data and materials will be monitored every year by an independent monitor.

### Safety

**Fig. 3:**
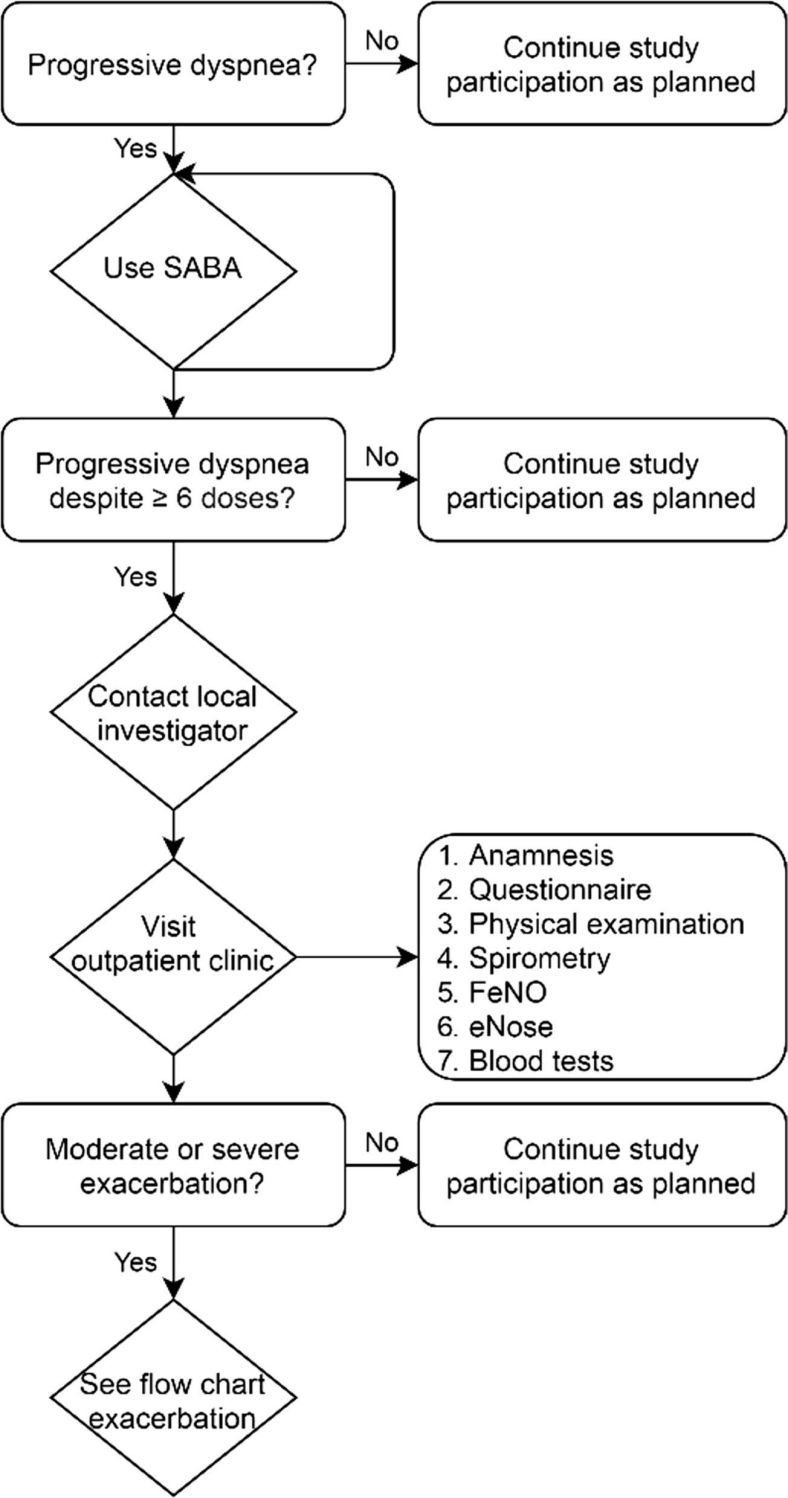
Flowchart for subjects with progressive dyspnea

**Fig. 4:**
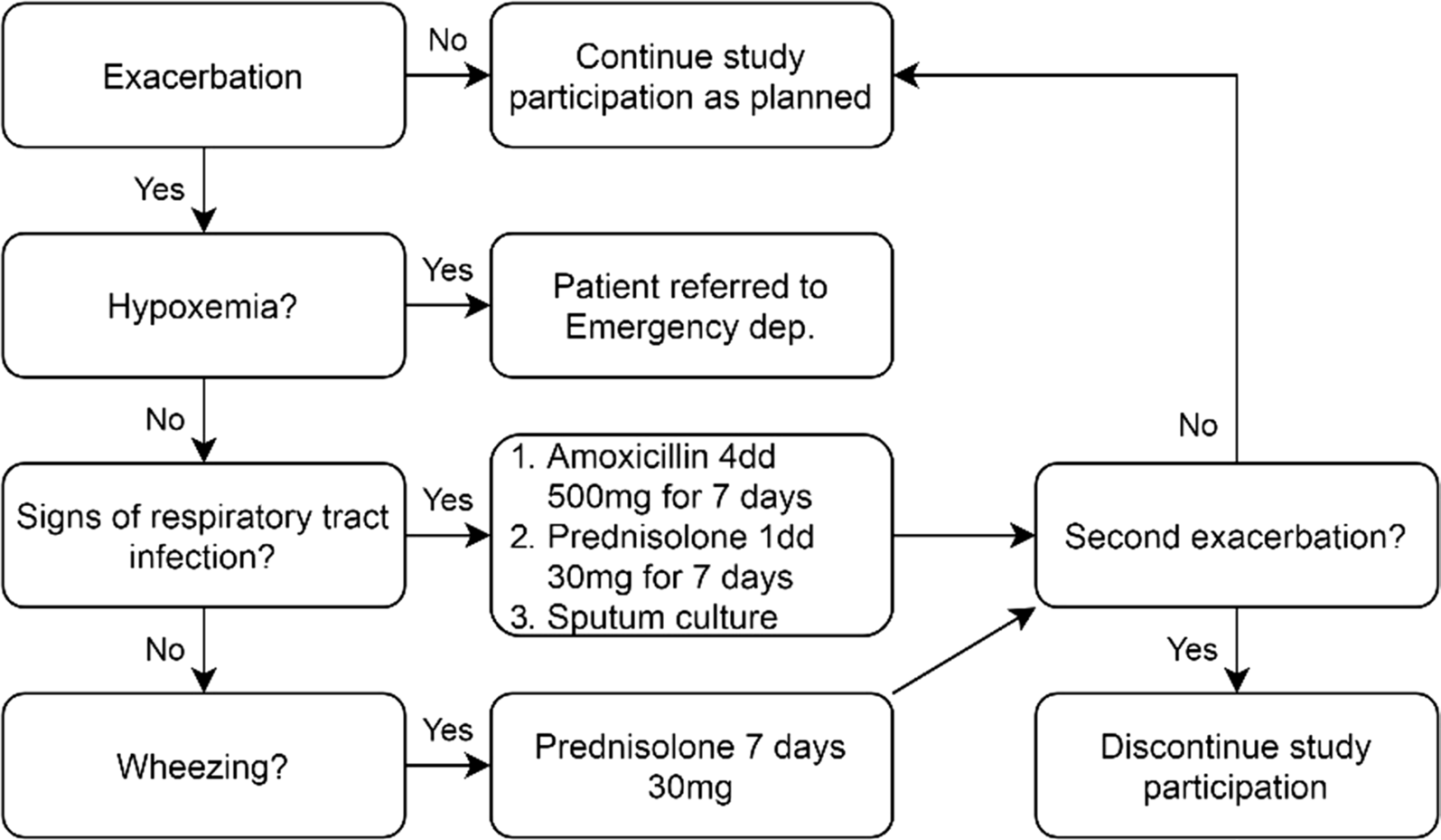
Flowchart for subjects with a moderate or severe exacerbation

Safety is assured by close monitoring of the subjects using a weekly questionnaire and online diary. Additionally, the use of reliever medication (salbutamol) is monitored to keep track of asthma control. Loss of asthma control is defined as an increase in ACQ score of more than 0.5 points for at least 4 days or progressive dyspnea despite using 500mcg of salbutamol per day by pressurized metered-dose inhaler (pMDI) with spacer. Patients will be asked to contact the coordinating researcher in case of progressive dyspnea despite using ≥ 600mcg salbutamol by pMDI with spacer. A central telephone number (the STOP-telephone) is available for all patients included in this study. As a backup, patients may email (STOP-mail). As a second backup, the local investigators and the general practitioner will be notified of the subject’s study participation and supplied with helpful flowcharts (see Figs. 3 and 4). We aim to follow this procedure before any antibiotics, OCS or ICS are given unless acute treatment is deemed necessary. Patients receive usual care during exacerbation. In case of a second moderate or severe exacerbation (e.g. not related to the first exacerbation), patients are censored and will continue with usual care. It is important to note that patients may choose to stop their participation at any moment, as recommended by the ethical regulations. Study insurance ensures compensation for those who suffer harm from this trial, as regulated by Dutch law (WMO: Medical Research Involving Human Subjects Act) [38].

## Sample size and statistical analysis

### Sample size calculation

**Fig. 5:**
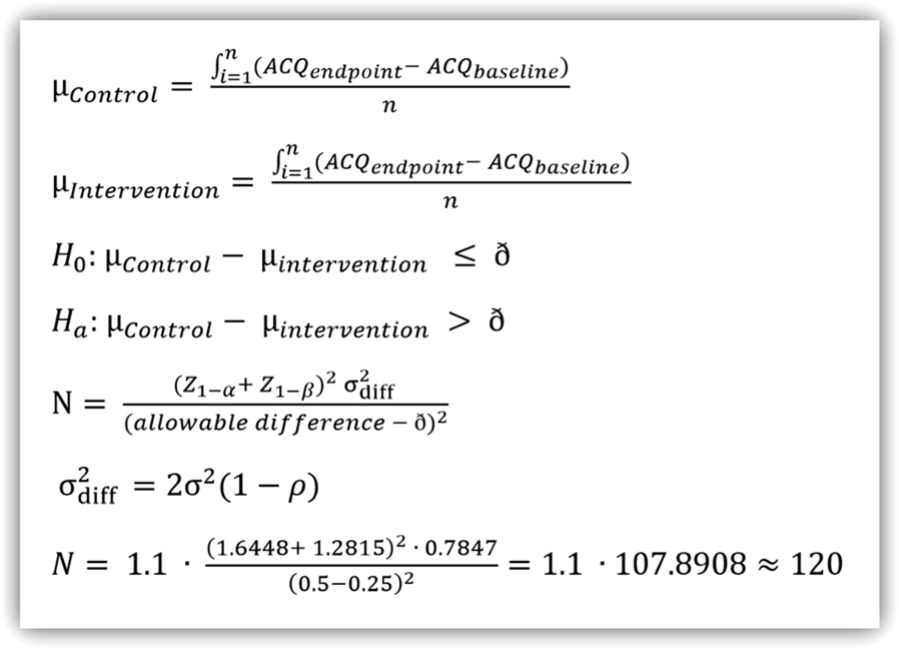
Formulae used in sample size calculation

The primary endpoint is the mean difference in ACQ score between the intervention period and the control period whit correction for the baseline ACQ score. This will be tested using a paired t-test for within-subjects designs, with a one-sided alpha of 0.05, a beta of 0.10, a rho of 0.5, a non-inferiority margin of 5% (0.25 ACQ points), an allowable difference (a minimal clinically important difference) of 0.5 ACQ points and a drop-out rate of 10%. The population variance ${\upsigma }_{\mathrm{e }}^{2}$ was calculated using a real live cohort of patients in the Franciscus Gasthuis & Vlietland. This cohort included all new referrals at the outpatient clinic with a suspicion of asthma. This cohort (n = 52) was selected for having a BMI ≥ 30, blood eosinophils ≤ 0.15 cells/µl, clinically proven asthma, and no obstructive lung diseases otherwise. All patients filled in an ACQ at baseline and 6 months, after which the difference ${ACQ}_{endpoint}- {ACQ}_{baseline}$ was calculated for every patient. This resulted in a mean of − 0.2749 with a variance 0.7847. A sample size of 120 patients was calculated using the formulae in Fig. 5.

### Statistical analysis

Study data will be analysed according to the intention-to-treat approach. Results of the statistical analysis consist of descriptive univariable and multivariable analysis. Continuous parameters with (approximately) normal distributions will be summarized with means and SDs (or 95% CIs). Continuous primary study parameters with skewed distributions will be summarized with medians and interquartile ranges (IQR). Finally, nominal and ordinal primary study parameters will be summarized with numbers (percentages).

1. **Primary study parameter**

The primary endpoint is defined as the difference in ACQ between the intervention period and control period relative to the baseline ACQ score. Definitions:

- Baseline: ACQ score at the first visit of period 1 or period 2
- Endpoint: ACQ at last visit of period 1 or period 2

The non-inferiority margin is 5% (i.e., 0.25 out of a maximum of 5 ACQ points). Non-inferiority will be established if the lower limit of a one-sided 95% confidence interval for the mean difference in ACQ score between the intervention period and control period is within the absolute 5% non-inferiority margin of the mean in the control arm.

2. Secondary study parameter(s)

The time to first exacerbation will be tested with Kaplan–Meier curves and log-rank tests to estimate the effect over time for both patient groups. Next, Cox regression will be used to measure the treatment effect adjusted for BMI and hospital as fixed covariates.

The majority of secondary variables are continuous and will be described by descriptive compared between intervention group and control group.

The change in time of several secondary parameters will be estimated by repeated measurements analysis. Linear mixed modeling will be used for continuous variables. Generalized estimated equations for binary data with logit link function will be used for the categorical variables.

3. Interim analysis

No interim analysis has been planned to prevent alpha loss. An independent Data Safety Monitoring Board (DSMB) may conduct an investigation of adverse events when ≥ 12 exacerbations may have occurred.

## Discussion

Obesity is more and more present among all asthma endotypes, often associated with T2-low endotypes and therefore not responsive to steroids. Nevertheless, guidelines advocate the use of ICS in all asthma patients, regardless of T2-endotype. As a result, obese T2-low asthma patients potentially suffer from the side effects of high dosed ICS while remaining poorly controlled.

T2 low asthma can be classified as obesity-related asthma, paucigranulocytic asthma or neutrophilic asthma with overlap between phenotypes [3, 39]. With the obesity pandemic soaring, the prevalence of asthma in combination with obesity is increasing, resulting in poor outcomes, such as a high burden of disease, frequent exacerbations, and increased risk for hospitals admission [40–44]. In this group, approximately 75% of patients can be classified as uncontrolled [45]. In patients with evident type 2 inflammation, such as eosinophilic- or allergic disease, good asthma control is often achieved by treatment with corticosteroids. However, T2-low asthma differs substantially from T2-high asthma, as underlying pathways are steroid-resistant [46–50]. Still, GINA guidelines recommend escalating ICS dose in patients with poor asthma control, without considering the T2-endotype. This is potentially problematic in patients with T2-low asthma, as these patients typically suffer from a high disease burden, regardless of ICS dose. This may lead to high dosed ICS without substantial improvement of asthma control [51]. High dosed ICS subsequently increases the risk of side effects, such as weight gain, diabetes, and high blood pressure [52–54]. These side effects are unfavourable, particularly in patients with the common combination of T2-low asthma and obesity, as high dosed ICS may worsen obesity, subsequently leading to worse asthma control, creating a vicious circle. This hypothesis is supported by a recent trial that demonstrated that asthma control decreased after increasing the dose of ICS in patients with non-eosinophilic or neutrophilic sputum [45]. We postulate that the adverse effects of ICS do not outweigh the health benefits in these patients.

The STOP trial is the first to investigate ICS tapering and discontinuation in the population of patients with T2-low asthma and obesity. Several trials on ICS tapering have been published in the past decades [15–21, 51, 55, 56]. However, these trials did not differentiate between T2-high and T2-low asthma. This concludes that ICS cannot be tapered in a mixed population of T2-high and T2-low asthma. However, additional subgroup analysis found that patients with low eosinophil count were more likely to taper and/or discontinue ICS [18, 20, 55]. Later studies, such as Demarche et al., selected patients with low eosinophils and successfully tapering or discontinuing ICS in more than half of these patients. Those encouraging results demonstrated the importance of selection on low blood eosinophils. In addition, recent guidelines usually define the threshold of low blood eosinophils as < 150 cells/µL, whereas earlier trials used thresholds of < 300-400cells/µL. Thus, adhering to the most recent definition of low blood eosinophils may increase the percentage of success.

The primary safety issue regarding this trial consists of patients worsening due to ICS tapering. Based on earlier trials and literature, we estimate the risk of severe exacerbations in patients with obese T2-low asthma as very low in patients with T2-low asthma and obesity [15, 17, 56]. However, to meet safety standards, a detailed plan was created to handle these adverse events.

Current guidelines advocate the use of ICS in all asthma patients. The STOP trial investigates opportunities to taper ICS to zero in patients with obese T2 low asthma. Successful discontinuation of ICS will lead to decreased exposure to glucocorticosteroids. On the contrary, failed ICS tapering justifies exposure to glucocorticosteroids in this patients group, as benefits appear to outweigh risks. Either way, the outcomes of this trial are expected to influence future guidelines on T2 low asthma and obesity.

## Data Availability

Data for this study are not yet available. After completion of the STOP trial, raw data can be obtained upon reasonable request from the corresponding author.

## Abbreviations

ACQ: Asthma Control Questionnaire
AQLQ: Asthma Quality of Life Questionnaire
BMI: Body Mass Index
CCMO: Dutch, competent authority
CI: Confidence interval
COPD: Chronic obstructive pulmonary disease
CGG: Dutch, obesity clinic CGG
DSMB: Data Safety Monitoring Board
EDC: Electronic data capturing
eNose: Electronic nose
FeNO: Fractional exhaled nitric oxide
FEV1: Forced exhaled volume in 1 s
FSS: Fatigue Severity Scale
GCP: Good clinical practice
ICS: Inhaled CorticoSteroids
IMDH: Inosine monophosphate dehydrogenase
IQR: InterQuartile range
MRC: Medical Research Council
mTOR: Mechanistic target of rapamycin
NTR: Netherlands trial register
OCS: Oral CorticoSteroids
OSAS: Obstructive sleep apnea syndrome
PBMCs: Peripheral blood mononuclear cells
pMDI: Pressurized Metered-Dose inhaler
RCT: Randomized controlled trial
(S)AE: (Serious) adverse event
SD: Standard deviation
STOP: Steroid tapering in obese patients with type 2 low asthma
SUSAR: Suspected unexpected adverse reaction
T2-hidden: Type 2 hidden
T2-high: Type 2 high
T2-low: Type 2 low
WMO: Dutch, Medical Research Involving Human Subjects Act
